# Reported recommendations to address cardiovascular risk factors differ by socio-economic status in Brazil. Results from the Brazilian National Health Survey 2019

**DOI:** 10.1016/j.pmedr.2023.102527

**Published:** 2023-11-25

**Authors:** Pollyanna Patriota, Ko Ko Maung, Pedro Marques-Vidal

**Affiliations:** a Pôle Santé Vallée de Joux – Joux Valley Health Center, Le Sentier, Switzerland; b Department of Medicine, Internal Medicine, Lausanne University Hospital and University of Lausanne, Lausanne, Switzerland

**Keywords:** Lifestyle recommendations, Epidemiology, Cardiovascular risk factor, Dietary recommendations, Socio-economic status

## Abstract

**Background:**

Management of cardiovascular risk factors (high cholesterol, diabetes, and hypertension) should start by implementing a healthy lifestyle. Whereas lifestyle recommendations are provided irrespective of the patient’s socio-economic status has not been recently assessed in the Brazilian population.

**Aims:**

To assess the preventive measures against cardiovascular risk factors according to educational level and income in the Brazilian population.

**Methods:**

Survey data of the 2019 Brazilian National Health Survey (PNS). The PNS is a nationwide household-based survey carried out by the Brazilian Ministry of Health. The PNS included face-to-face interviews and collected information on lifestyle management of high cholesterol, diabetes, and hypertension by a healthy diet, an adequate weight, exercise, and quitting smoking. The participant’s educational level and income (in multiples of the basic salary per capita) was collected.

**Results:**

Of the 88,052 participants included, 13,151 (14.9%), 6,986 (7.9%) and 22,516 (25.6%) reported being diagnosed with high cholesterol, diabetes, or hypertension, respectively. Dietary recommendations were the most frequently provided (94.5%, 94.6% and 88.1% for high cholesterol diabetes, and hypertension, respectively), while recommendations to quit smoking to current smokers were the least frequently provided (74.9%, 85.8% and 81.1% for high cholesterol, diabetes, and hypertension, respectively). After multivariable adjustment, participants with a higher educational level or a higher income had a higher likelihood of reporting receipt lifestyle recommendations for high cholesterol or hypertension, while no associations were found for most recommendations for diabetes.

**Conclusion:**

Better-educated, wealthier Brazilians report receiving more lifestyle recommendations regarding high cholesterol and hypertension management more frequently than lower-educated or with low-income.

## 1 Introduction

Cardiovascular diseases (CVD) rank among the leading causes of death from noncommunicable diseases (NCDs) worldwide (GBD, 2016). Brazil is the fifth largest country in terms of population (207 million in 2022) and CVD represents almost one-third (31 %) of all deaths, affecting men more frequently than women (Mansur et al., 1996). In 2016, ischemic heart disease (IHD) was the leading cause of years of life lost (YLL) in Brazil (GBD, 2016). Even though, from 1996 to 2019, a significant reduction in the mortality rate due to CVD, IHD and stroke was observed (Mansur et al., 1996, Dai et al., 2017).

The main modifiable risk factors for CVD are obesity, hypertension, smoking, dyslipidaemia, and diabetes (Visseren et al., 2021). In Brazil, the main contributors to CVD are overweight, obesity and high systolic blood pressure, which were reported by 60.2 %, 25.9 % and 25.2 % of the adult population, respectively (Ferreira et al., 10AD, Brasil, 2021). It is also well recognized that changes in lifestyle, properly implemented, promote reductions in cardiovascular risk factors like of single-drug therapies (Marques-Vidal, 2020). Weight loss, dietary changes (i.e., increasing potassium and fibre intake), increased aerobic exercise and smoking cessation can significantly impact cardiovascular risk factor levels (Marques-Vidal, 2020).

Still, initiatives promoting a healthier lifestyle tend to be more effective in segments of society with higher schooling and economic levels, which have access to greater knowledge and resources to change behaviours and adopt healthier practices. Prevalence of NCDs varies according to educational level (Macinko and Mullachery, 2013), making social inequality, including low education and low income, a key factor in the burden of NCDs (Martinez et al., 1990). In Brazil, despite a decrease in some unhealthy behaviours, educational inequalities were still strongly associated with physical inactivity, low fruit and high sugary drinks consumption (Barros et al., 2013).

Whereas those inequalities can also be due to differences in the recommendations provided by doctors has seldom been studied. Hence, we aimed to assess in the Brazilian population the preventive lifestyle recommendations against cardiovascular risk factors reported by patients according to their educational level and income. As Brazil has a universal health coverage, our initial hypothesis was that lifestyle recommendations against CVD risk factors would be provided to all patients irrespective of their educational or income level.

## 2 Participants and methods

### 2.1 Study design

This cross-sectional study used data from the Brazilian National Health Survey (PNS): It is a five-year household survey with a complex sample design, representative of the Brazilian population in macro-regions, states, urban, rural, and metropolitan areas. The Brazilian Ministry of Health and the Brazilian Institute of Geography and Statistics (IBGE) developed and carried this study. Anonymized data are publicly available on the IBGE website (https://www. ibge.gov.br/). The PNS was approved by the Brazilian *Comissão Nacional de Ética em Pesquisa* (National Committee of Ethics in Research) in July 2013 (reference 328.159) and 2019 (reference 3.529.376) and participants provided informed consent before entering. Full details can be obtained at https://www.pns.icict.fiocruz.br/aspectos-eticos/.

The sampling process included three stages; in the first, the census tracts were drawn and, in the second, the households. In the third stage, one resident aged 15 years or more was drawn from each selected household. The questionnaire applied by the PNS is divided in three parts addressing characteristics of: (1) household, (2) all residents and (3) the selected resident. Details about the sampling process and methods of PNS 2019 are available in other publications (Stopa et al., 2019).

### 2.2 Diagnosis and management of cardiovascular risk factors

Participants were considered as presenting with high cholesterol, diabetes, or hypertension if they replied positively to the questions “Has a doctor diagnosed you with high cholesterol?”, “Has a doctor diagnosed you with diabetes?” and “Has a doctor diagnosed you with hypertension?”, respectively. In the case of a positive answer to any of the questions, a series of questions related to recommendations on healthy behaviours was applied. Four (healthy eating, maintaining a healthy weight, exercising regularly, and quitting smoking) were common to all three risk factors and were considered in this study. The original questions are indicated in supplementary Table S1.

### 2.3 Covariates

All covariates were obtained by questionnaire. We used gender (man and woman), ethnicity (white, black, Asian, mixed and native Brazilian), marital status (single, married, divorced and widowed), educational level [none, (in)complete basic, (in)complete secondary school and (in)complete university], smoking (never, former, current), and income expressed as multiples of the minimum salary (≤1, >1 to 3, >3 to 5 and > 5).

### 2.4 Exclusion criteria

Participants were excluded if 1) they had no diagnosis of dyslipidaemia, diabetes, and hypertension; 2) they presented with pregnancy-related hypertension or diabetes, and 3) they had missing data for covariates.

Ethical statement

The PNS project was approved by the National Research Ethics Committee (process No. 3.529,376, of August 23, 2019). All respondents signed an informed consent form.

### 2.5 Statistical analysis

Statistical analysis was conducted using Stata version 16.1 for Windows (Stata corp., College Station, TX; USA). Descriptive statistics were presented as number of participants (percentage) for categorical variables or as average ± standard deviation for continuous variables. Between-group comparisons were performed using chi-square or Fisher’s exact test for categorical variables and student’s *t*-test for continuous variables. Multivariable analysis of the variables associated with lifestyle recommendations was conducted using logistic regression and results were expressed as odds-ratio and (95 % CI).

Sensitivity analyses were conducted using inverse probability weighting to take into account excluded participants. The probability of inclusion was modelled using a logistic regression model and the inverse of the probability was used as weighting in the multivariable analyses (Narduzzi et al., 2014). Due to sample size and the number of statistical tests performed, statistical significance was considered for a two-sided test with p < 0.001.

## 3 Results

### 3.1 Characteristics of participants

**Fig. 1:**
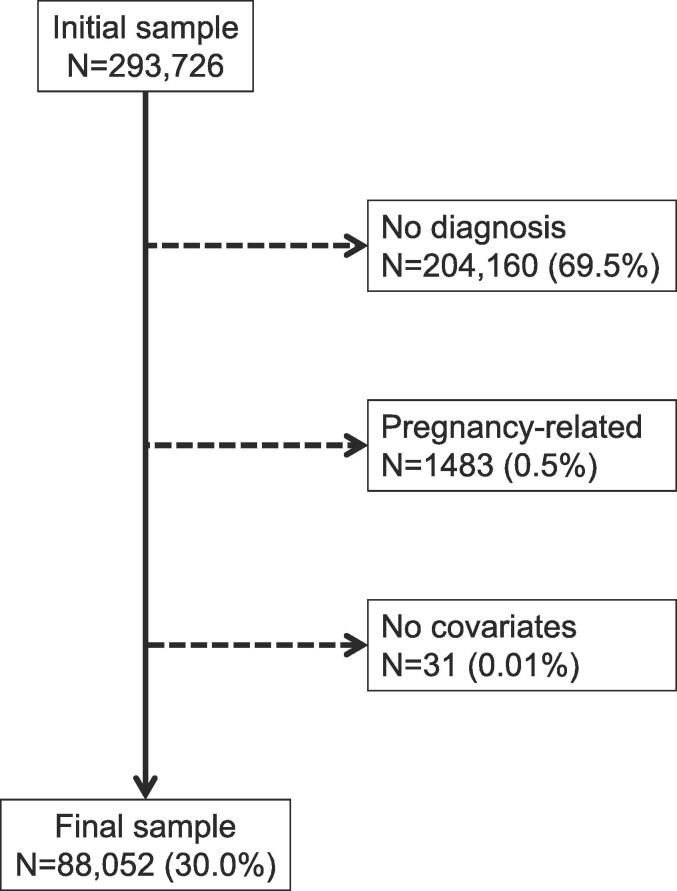

Of the initial 293,726 participants, 88,052 (30.0 %) with at least one CVD risk factor diagnosed were included. The reasons for exclusion are indicated in Fig. 1 and the characteristics of the included and the excluded participants are provided in supplementary Table S2. Included participants were more frequently women, older, less frequently of mixed ethnicity or single, more highly educated and less frequently smokers.

### 3.2 Factors associated with provision of lifestyle recommendations for high cholesterol

Of the 88,052 participants, 13,151 (14.9 %) reported being diagnosed with high cholesterol. Among those, 12,427 (94.5 %) reported having received recommendations regarding healthy eating, 11,756 (89.4 %) to maintain a healthy weight, and 11,510 (87.5 %) to exercise regularly. Among the 1,305 current smokers, 978 (74.9 %) reported being advised to quit smoking. The factors associated with lifestyle recommendations towards high cholesterol are summarized in Table 1 for bivariate analyses and in Table 2 for multivariable analyses. Overall, participants receiving lifestyle recommendations were younger, more frequently married, more frequently of black ethnicity, more frequently non-smokers, and with a higher income. Those findings were maintained after accounting for exclusions **(**supplementary Table 3).

**Table 1:** Association between sociodemographic characteristics and recommendations provided reported by adult participants diagnosed with high cholesterol, brazilian national health survey 2019.

	Healthy eating			Healthy weight			Regular physical activity		
	Yes	No	P-value	Yes	No	P-value	Yes	No	P-value
Sample size	12,427	724		11,756	1395		11,510	1641	
Women (%)	8,014 (64.5)	482 (66.6)	0.254	7,548 (64.2)	948 (68)	0.006	7,402 (64.3)	1,094 (66.7)	0.062
Age (years)	56.1 ± 15	58.5 ± 14.1	<0.001	56.2 ± 14.8	56.5 ± 16.1	0.488	55.6 ± 14.8	60.7 ± 15.7	<0.001
Ethnicity (%)			0.098			0.067			0.024
White	5,036 (40.5)	281 (38.8)		4,758 (40.5)	559 (40.1)		4,705 (40.9)	612 (37.3)	
Black	1,365 (11.0)	65 (9.0)		1,306 (11.1)	124 (8.9)		1,256 (10.9)	174 (10.6)	
Asian	109 (0.9)	11 (1.5)		105 (0.9)	15 (1.1)		109 (1)	11 (0.7)	
Mixed	5,831 (46.9)	363 (50.1)		5,504 (46.8)	690 (49.5)		5,363 (46.6)	831 (50.6)	
Native	86 (0.7)	4 (0.6)		83 (0.7)	7 (0.5)		77 (0.7)	13 (0.8)	
Marital status (%)			0.367			<0.001			<0.001
Married	5,485 (44.1)	296 (40.9)		5,243 (44.6)	538 (38.6)		5,162 (44.9)	619 (37.7)	
Divorced	1,378 (11.1)	86 (11.9)		1,314 (11.2)	150 (10.8)		1,292 (11.2)	172 (10.5)	
Widowed	1,904 (15.3)	113 (15.6)		1,804 (15.4)	213 (15.3)		1,671 (14.5)	346 (21.1)	
Single	3,660 (29.5)	229 (31.6)		3,395 (28.9)	494 (35.4)		3,385 (29.4)	504 (30.7)	
Smoking status (%)			0.001			<0.001			<0.001
Never	6,734 (54.2)	347 (47.9)		6,386 (54.3)	695 (49.8)		6,283 (54.6)	798 (48.6)	
Former	4,483 (36.1)	282 (39.0)		4,248 (36.1)	517 (37.1)		4,130 (35.9)	635 (38.7)	
Current	1,210 (9.7)	95 (13.1)		1,122 (9.5)	183 (13.1)		1,097 (9.5)	208 (12.7)	
Educational level (%)			<0.001			<0.001			<0.001
None	1,304 (10.5)	102 (14.1)		1,212 (10.3)	194 (13.9)		1,089 (9.5)	317 (19.3)	
Basic	5,449 (43.9)	389 (53.7)		5,123 (43.6)	715 (51.3)		4,920 (42.8)	918 (55.9)	
Secondary	3,204 (25.8)	133 (18.4)		3,049 (25.9)	288 (20.7)		3,087 (26.8)	250 (15.2)	
University	2,470 (19.9)	100 (13.8)		2,372 (20.2)	198 (14.2)		2,414 (21)	156 (9.5)	
Income level (%)			<0.001			<0.001			<0.001
Till 1 salary	5,841 (47)	419 (57.9)		5,472 (46.6)	788 (56.5)		5,302 (46.1)	958 (58.4)	
>1 to 3 salaries	4,570 (36.8)	228 (31.5)		4,328 (36.8)	470 (33.7)		4,258 (37)	540 (32.9)	
>3 to 5 salaries	999 (8.0)	36 (5.0)		962 (8.2)	73 (5.2)		960 (8.3)	75 (4.6)	
>5 salaries	1,017 (8.2)	41 (5.7)		994 (8.5)	64 (4.6)		990 (8.6)	68 (4.1)	

	Stop smoking^a^		
	Yes	No	P-value
Sample size	978	327	
Women (%)	554 (56.7)	187 (57.2)	0.864
Age (years)	55.1 ± 12.1	52.4 ± 13.6	<0.001
Ethnicity (%)			§ 0.330
White	378 (38.7)	121 (37.0)	
Black	138 (14.1)	34 (10.4)	
Asian	10 (1.0)	3 (0.9)	
Mixed	443 (45.3)	165 (50.5)	
Native	9 (0.9)	4 (1.2)	
Marital status (%)			0.127
Married	334 (34.2)	102 (31.2)	
Divorced	147 (15.0)	39 (11.9)	
Widowed	133 (13.6)	41 (12.5)	
Single	364 (37.2)	145 (44.3)	
Educational level (%)			0.021
None	104 (10.6)	52 (15.9)	
Basic	500 (51.1)	174 (53.2)	
Secondary	213 (21.8)	55 (16.8)	
University	161 (16.5)	46 (14.1)	
Income level (%)			0.024
Till 1 salary	498 (50.9)	192 (58.7)	
>1 to 3 salaries	332 (34.0)	103 (31.5)	
>3 to 5 salaries	72 (7.4)	19 (5.8)	
>5 salaries	76 (7.8)	13 (4.0)	

Results are expressed as number of participants (column percentage) for categorical variables and mean ± standard deviation for continuous variables.

a among current smokers only. Between-group comparisons performed using chi-square or Fisher’s exact test (§) for categorical variables and by student’s *t*-test for continuous variables.

**Table 2:** Multivariable association between sociodemographic characteristics and recommendations provided reported by adult participants diagnosed with high cholesterol, brazilian national health survey 2019.

	Healthy eating	P-value	Healthy weight	P-value	Physical activity	P-value	Stop smoking^a^	P-value
Women	0.95 (0.80–1.12)	0.555	0.89 (0.78–1.01)	0.065	1.05 (0.93–1.18)	0.420	0.97 (0.74–1.28)	0.839
Age (per decade)	0.90 (0.84–0.96)	0.001	1.00 (0.95–1.04)	0.880	0.83 (0.79–0.87)	<0.001	1.23 (1.10–1.39)	<0.001
Ethnicity								
White	1 (ref)		1 (ref)		1 (ref)		1 (ref)	
Black	1.38 (1.04–1.82)	0.026	1.53 (1.24–1.89)	<0.001	1.19 (0.99–1.43)	0.070	1.62 (1.03–2.53)	0.035
Asian	0.56 (0.30–1.06)	0.073	0.82 (0.47–1.43)	0.481	1.33 (0.71–2.52)	0.374	1.03 (0.27–3.93)	0.960
Mixed	1.02 (0.86–1.21)	0.809	1.12 (0.99–1.27)	0.077	0.99 (0.88–1.12)	0.885	1.04 (0.78–1.39)	0.799
Native	1.39 (0.50–3.83)	0.524	1.69 (0.77–3.68)	0.189	0.89 (0.49–1.63)	0.711	0.67 (0.20–2.27)	0.518
Marital status								
Married	1 (ref)		1 (ref)		1 (ref)		1 (ref)	
Divorced	0.88 (0.68–1.13)	0.300	0.90 (0.74–1.09)	0.280	0.86 (0.71–1.03)	0.107	1.01 (0.65–1.54)	0.981
Widowed	1.14 (0.89–1.45)	0.306	0.99 (0.83–1.19)	0.950	0.85 (0.72–0.99)	0.040	0.89 (0.57–1.41)	0.629
Single	0.83 (0.69–1.00)	0.055	0.72 (0.63–0.83)	<0.001	0.73 (0.64–0.83)	<0.001	0.86 (0.63–1.17)	0.330
Smoking status								
Never	1 (ref)		1 (ref)		1 (ref)		NC	
Former	0.90 (0.77–1.07)	0.233	0.93 (0.82–1.05)	0.255	1.00 (0.89–1.13)	0.965	NC	
Current	0.71 (0.56–0.90)	0.005	0.72 (0.60–0.86)	<0.001	0.76 (0.64–0.90)	0.002	NC	
Educational level								
None	0.67 (0.50–0.90)	0.009	0.62 (0.50–0.77)	<0.001	0.41 (0.33–0.50)	<0.001	0.41 (0.25–0.68)	<0.001
Basic	1 (ref)		1 (ref)		1 (ref)		1 (ref)	
Secondary	0.67 (0.54–0.83)	<0.001	0.71 (0.61–0.83)	<0.001	0.54 (0.46–0.63)	<0.001	0.70 (0.48–1.00)	0.052
University	0.89 (0.67–1.19)	0.432	0.95 (0.78–1.17)	0.634	1.06 (0.85–1.33)	0.601	0.78 (0.48–1.27)	0.316
P for trend	0.029		<0.001		<0.001		0.018	
Income level								
Till 1 salary	1 (ref)		1 (ref)		1 (ref)		1 (ref)	
>1 to 3 salaries	1.36 (1.14–1.62)	0.001	1.21 (1.06–1.37)	0.005	1.26 (1.12–1.43)	<0.001	1.07 (0.79–1.44)	0.671
>3 to 5 salaries	1.74 (1.19–2.53)	0.004	1.59 (1.21–2.09)	0.001	1.60 (1.23–2.09)	0.001	1.29 (0.69–2.41)	0.419
>5 salaries	1.55 (1.05–2.28)	0.026	1.85 (1.37–2.50)	<0.001	1.67 (1.24–2.24)	0.001	1.84 (0.91–3.74)	0.092
P for trend	0.015		<0.001		<0.001		0.086	

a among current smokers only. NC, not considered. Results are expressed as odds ratio and (95% confidence interval) for a positive answer regarding the provision of the recommendation. Individual odds ratios are adjusted on all other variables of the table. Statistical analysis by logistic regression.

### 3.3 Factors associated with provision of lifestyle recommendations for diabetes

Overall, 6,986 participants (7.9 % of the retained sample) reported being diagnosed with diabetes. Among those, 5,887 (94.6 %) reported having received recommendations regarding healthy eating, 5,725 (92.0 %) to maintain a healthy weight, and 5,335 (85.7 %) to exercise regularly. Among the 572 current smokers, 491 (85.8 %) reported being advised to quit smoking. The factors associated with lifestyle recommendations towards diabetes are summarized in Table 3 for bivariate analyses and in Table 4 for multivariable analyses. Overall, participants receiving lifestyle recommendations were younger, and with a higher income. Those findings were maintained after accounting for exclusions (supplementary Table 4).

**Table 3:** Association between sociodemographic characteristics and recommendations provided reported by adult participants diagnosed with diabetes, brazilian national health survey 2019.

	Healthy eating			Healthy weight			Regular physical activity		
	Yes	No	P-value	Yes	No	P-value	Yes	No	P-value
Sample size	5,887	337		5,725	499		5,335	889	
Women (%)	3,496 (59.4)	214 (63.5)		3,404 (59.5)	306 (61.3)	0.416	3,156 (59.2)	554 (62.3)	0.075
Age (years)	61.9 ± 12.9	64.1 ± 12.7	0.003	61.8 ± 12.9	64.5 ± 13.1	<0.001	61.2 ± 12.7	66.9 ± 12.9	<0.001
Ethnicity (%)			§ 0.718						0.129
White	2,262 (38.4)	123 (36.5)		2,203 (38.5)	182 (36.5)		2,027 (38.0)	358 (40.3)	
Black	739 (12.6)	39 (11.6)		719 (12.6)	59 (11.8)		677 (12.7)	101 (11.4)	
Asian	59 (1)	2 (0.6)		56 (1.0)	5 (1.0)		58 (1.1)	3 (0.3)	
Mixed	2,780 (47.2)	171 (50.7)		2,699 (47.1)	252 (50.5)		2,529 (47.4)	422 (47.5)	
Native	47 (0.8)	2 (0.6)		48 (0.8)	1 (0.2)		44 (0.8)	5 (0.6)	
Marital status (%)			0.144			0.249			<0.001
Married	2,580 (43.8)	126 (37.4)		2,509 (43.8)	197 (39.5)		2,357 (44.2)	349 (39.3)	
Divorced	644 (10.9)	42 (12.5)		632 (11.0)	54 (10.8)		602 (11.3)	84 (9.5)	
Widowed	1,186 (20.2)	76 (22.6)		1,152 (20.1)	110 (22.0)		1,021 (19.1)	241 (27.1)	
Single	1,477 (25.1)	93 (27.6)		1,432 (25)	138 (27.7)		1,355 (25.4)	215 (24.2)	
Smoking status (%)			0.164			0.039			0.002
Never	2,997 (50.9)	154 (45.7)		2,920 (51.0)	231 (46.3)		2,750 (51.6)	401 (45.1)	
Former	2,354 (40)	147 (43.6)		2,292 (40.0)	209 (41.9)		2,103 (39.4)	398 (44.8)	
Current	536 (9.1)	36 (10.7)		513 (9.0)	59 (11.8)		482 (9.0)	90 (10.1)	
Educational level (%)			0.002			<0.001			<0.001
None	920 (15.6)	71 (21.1)		868 (15.2)	123 (24.7)		753 (14.1)	238 (26.8)	
Basic	3,038 (51.6)	186 (55.2)		2,961 (51.7)	263 (52.7)		2,735 (51.3)	489 (55.0)	
Secondary	1,242 (21.1)	50 (14.8)		1,221 (21.3)	71 (14.2)		1,185 (22.2)	107 (12.0)	
University	687 (11.7)	30 (8.9)		675 (11.8)	42 (8.4)		662 (12.4)	55 (6.2)	
Income level (%)			0.002			<0.001			<0.001
Till 1 salary	2,944 (50)	205 (60.8)		2,851 (49.8)	298 (59.7)		2,630 (49.3)	519 (58.4)	
>1 to 3 salaries	2,177 (37)	100 (29.7)		2,120 (37)	157 (31.5)		1,972 (37)	305 (34.3)	
>3 to 5 salaries	446 (7.6)	18 (5.3)		439 (7.7)	25 (5.0)		424 (8.0)	40 (4.5)	
>5 salaries	320 (5.4)	14 (4.2)		315 (5.5)	19 (3.8)		309 (5.8)	25 (2.8)	

	Stop smoking^a^		
	Yes	No	P-value
Sample size	491	81	
Women (%)	246 (50.1)	51 (63.0)	0.032
Age (years)	58.5 ± 11.8	57.5 ± 13.5	0.475
Ethnicity (%)			§ 0.114
White	174 (35.4)	32 (39.5)	
Black	71 (14.5)	5 (6.2)	
Asian	9 (1.8)	0 (0)	
Mixed	232 (47.3)	42 (51.9)	
Native	5 (1.0)	2 (2.5)	
Marital status (%)			0.601
Married	164 (33.4)	32 (39.5)	
Divorced	76 (15.5)	10 (12.4)	
Widowed	86 (17.5)	11 (13.6)	
Single	165 (33.6)	28 (34.6)	
Educational level (%)			0.794
None	82 (16.7)	16 (19.8)	
Basic	269 (54.8)	41 (50.6)	
Secondary	90 (18.3)	17 (21.0)	
University	50 (10.2)	7 (8.6)	
Income level (%)			§ 0.741
Till 1 salary	266 (54.2)	48 (59.3)	
>1 to 3 salaries	166 (33.8)	27 (33.3)	
>3 to 5 salaries	26 (5.3)	3 (3.7)	
>5 salaries	33 (6.7)	3 (3.7)	

a Among current smokers only. Results are expressed as number of participants (column percentage) for categorical variables and mean ± standard deviation for continuous variables. Between-group comparisons performed using chi-square or Fisher’s exact test (§) for categorical variables and by student’s *t*-test for continuous variables.

**Table 4:** Multivariable association between sociodemographic characteristics and recommendations provided reported by adult participants diagnosed with diabetes, brazilian national health survey 2019.

	Healthy eating	P-value	Healthy weight	P-value	Physical activity	P-value	Stop smoking^a^	P-value
Women	0.89 (0.69–1.13)	0.330	0.97 (0.79–1.18)	0.750	0.95 (0.81–1.12)	0.567	0.54 (0.32–0.91)	0.021
Age (per decade)	0.86 (0.78–0.95)	0.004	0.84 (0.77–0.91)	<0.001	0.70 (0.65–0.75)	<0.001	1.06 (0.84–1.33)	0.626
Ethnicity								
White	1 (ref)		1 (ref)		1 (ref)		1 (ref)	
Black	1.15 (0.79–1.68)	0.473	1.14 (0.83–1.56)	0.414	1.34 (1.05–1.72)	0.019	2.71 (0.99–7.38)	0.051
Asian	1.52 (0.36–6.31)	0.568	0.86 (0.34–2.18)	0.745	2.95 (0.91–9.61)	0.072	NA	
Mixed	0.97 (0.75–1.24)	0.785	0.98 (0.79–1.20)	0.812	1.16 (0.99–1.36)	0.075	1.05 (0.62–1.78)	0.852
Native	1.50 (0.36–6.27)	0.582	4.65 (0.64–34.1)	0.130	1.78 (0.68–4.63)	0.237	0.45 (0.08–2.51)	0.364
Marital status								
Married	1 (ref)		1 (ref)		1 (ref)		1 (ref)	
Divorced	0.75 (0.52–1.08)	0.120	0.89 (0.65–1.23)	0.486	0.98 (0.76–1.28)	0.908	1.50 (0.68–3.29)	0.310
Widowed	0.94 (0.68–1.29)	0.694	1.05 (0.80–1.37)	0.724	0.95 (0.78–1.17)	0.632	1.86 (0.84–4.12)	0.126
Single	0.77 (0.58–1.03)	0.074	0.79 (0.62–1.00)	0.054	0.82 (0.67–0.99)	0.040	1.35 (0.75–2.45)	0.318
Smoking status								
Never	1 (ref)		1 (ref)		1 (ref)		NC	
Former	0.87 (0.68–1.10)	0.231	0.94 (0.77–1.15)	0.532	0.86 (0.73–1.00)	0.056	NC	
Current	0.77 (0.53–1.13)	0.177	0.69 (0.51–0.94)	0.019	0.72 (0.56–0.93)	0.013	NC	
Educational level								
None	0.70 (0.47–1.06)	0.089	0.55 (0.40–0.77)	0.001	0.47 (0.36–0.62)	<0.001	0.88 (0.38–2.00)	0.754
Basic	1 (ref)		1 (ref)		1 (ref)		1 (ref)	
Secondary	0.76 (0.55–1.06)	0.111	0.77 (0.58–1.02)	0.065	0.66 (0.53–0.84)	<0.001	1.19 (0.62–2.28)	0.597
University	0.82 (0.50–1.33)	0.418	0.83 (0.55–1.26)	0.376	0.94 (0.66–1.34)	0.722	1.02 (0.37–2.83)	0.968
P for trend	0.394		0.034		<0.001		0.874	
Income level								
Till 1 salary	1 (ref)		1 (ref)		1 (ref)		1 (ref)	
>1 to 3 salaries	1.48 (1.14–1.92)	0.003	1.35 (1.09–1.68)	0.005	1.29 (1.10–1.52)	0.002	1.08 (0.62–1.86)	0.796
>3 to 5 salaries	1.66 (0.97–2.82)	0.063	1.70 (1.08–2.67)	0.022	1.96 (1.36–2.83)	<0.001	1.47 (0.38–5.66)	0.579
>5 salaries	1.53 (0.82–2.86)	0.182	1.62 (0.95–2.78)	0.078	2.22 (1.39–3.53)	0.001	1.67 (0.42–6.66)	0.470
P for trend	0.174		0.057		<0.001		0.419	

a Among current smokers only. NA, not available; NC, not considered. Results are expressed as odds ratio and (95% confidence interval) for a positive answer regarding the provision of the recommendation. Individual odds ratios are adjusted on all other variables of the table. Statistical analysis by logistic regression.

### 3.4 Factors associated with provision of lifestyle recommendations for hypertension

Overall, 22,516 participants (25.6 % of the retained sample) reported being diagnosed with hypertension. Among those, 16,804 (88.1 %) reported having received recommendations regarding healthy eating, 16,304 (85.5 %) to maintain a healthy weight, and 15,635 (82.0 %) to exercise regularly. Among the 2,016 current smokers, 1,635 (81.1 %) reported being advised to quit smoking. The factors associated with lifestyle recommendations towards hypertension are summarized in Table 5 for bivariate analyses and in Table 6 for multivariable analyses. Overall, participants receiving lifestyle recommendations were younger, more frequently married, more frequently non-smokers, of a higher educational level, and with a higher income. Those findings were maintained after accounting for exclusions (supplementary Table 5).

**Table 5:** Association between sociodemographic characteristics and recommendations provided reported by adult participants diagnosed with hypertension, brazilian national health survey 2019.

	Healthy eating			Healthy weight			Regular physical activity		
	Yes	No	P-value	Yes	No	P-value	Yes	No	P-value
Sample size	16,804	2,272		16,304	2,772		15,635	3,441	
Women (%)	10,040 (59.8)	1,313 (57.8)	0.074	9,738 (59.7)	1,615 (58.3)	0.146	9,323 (59.6)	2,030 (59)	0.492
Age (years)	60.1 ± 14.1	61.0 ± 14.6	0.004	59.9 ± 14.1	61.8 ± 14.9	<0.001	59.4 ± 13.9	63.9 ± 14.9	<0.001
Ethnicity (%)			0.148			0.028			0.039
White	6,350 (37.8)	841 (37)		6,201 (38.0)	990 (35.7)		5,959 (38.1)	1,232 (35.8)	
Black	2,196 (13.1)	267 (11.8)		2,124 (13.0)	339 (12.2)		2,033 (13.0)	430 (12.5)	
Asian	135 (0.8)	17 (0.8)		130 (0.8)	22 (0.8)		128 (0.8)	24 (0.7)	
Mixed	7,997 (47.6)	1,135 (50.0)		7,727 (47.4)	1,405 (50.7)		7,403 (47.4)	1,729 (50.3)	
Native	126 (0.8)	12 (0.5)		122 (0.8)	16 (0.6)		112 (0.7)	26 (0.8)	
Marital status (%)			0.007			<0.001			<0.001
Married	7,370 (43.9)	937 (41.2)		7,205 (44.2)	1,102 (39.8)		6,953 (44.5)	1,354 (39.4)	
Divorced	1,750 (10.4)	237 (10.4)		1,691 (10.4)	296 (10.7)		1,652 (10.6)	335 (9.7)	
Widowed	3,185 (19.0)	413 (18.2)		3,042 (18.7)	556 (20.1)		2,790 (17.8)	808 (23.5)	
Single	4,499 (26.8)	685 (30.2)		4,366 (26.8)	818 (29.5)		4,240 (27.1)	944 (27.4)	
Smoking status (%)			<0.001			<0.001			<0.001
Never	9,026 (53.7)	1,018 (44.8)		8,802 (54.0)	1,242 (44.8)		8,423 (53.9)	1,621 (47.1)	
Former	6,107 (36.3)	909 (40.0)		5,905 (36.2)	1,111 (40.1)		5,672 (36.3)	1,344 (39.1)	
Current	1,671 (9.9)	345 (15.2)		1,597 (9.8)	419 (15.1)		1,540 (9.9)	476 (13.8)	
Educational level (%)			<0.001			<0.001			<0.001
None	2,421 (14.4)	425 (18.7)		2,256 (13.8)	590 (21.3)		1,988 (12.7)	858 (24.9)	
Basic	8,288 (49.3)	1,248 (54.9)		8,024 (49.2)	1,512 (54.6)		7,645 (48.9)	1,891 (55.0)	
Secondary	3,753 (22.3)	397 (17.5)		3,697 (22.7)	453 (16.3)		3,656 (23.4)	494 (14.4)	
University	2,342 (13.9)	202 (8.9)		2,327 (14.3)	217 (7.8)		2,346 (15.0)	198 (5.8)	
Income level (%)			<0.001			<0.001			<0.001
Till 1 salary	8,449 (50.3)	1,337 (58.9)		8,139 (49.9)	1,647 (59.4)		7,699 (49.2)	2,087 (60.7)	
>1 to 3 salaries	6,194 (36.9)	755 (33.2)		6,019 (36.9)	930 (33.6)		5,811 (37.2)	1,138 (33.1)	
>3 to 5 salaries	1,130 (6.7)	99 (4.4)		1,122 (6.9)	107 (3.9)		1,100 (7.0)	129 (3.8)	
>5 salaries	1,031 (6.1)	81 (3.6)		1,024 (6.3)	88 (3.2)		1,025 (6.6)	87 (2.5)	

	Stop smoking^a^		
	Yes	No	P-value
Sample size	1,635	381	
Women (%)	836 (51.1)	205 (53.8)	0.347
Age (years)	56.7 ± 12.8	58.1 ± 13.4	0.045
Ethnicity (%)			§ 0.177
White	533 (32.6)	106 (27.8)	
Black	267 (16.3)	57 (15.0)	
Asian	14 (0.9)	6 (1.6)	
Mixed	809 (49.5)	209 (54.9)	
Native	12 (0.7)	3 (0.8)	
Marital status (%)			0.163
Married	522 (31.9)	119 (31.2)	
Divorced	223 (13.6)	40 (10.5)	
Widowed	240 (14.7)	70 (18.4)	
Single	650 (39.8)	152 (39.9)	
Educational level (%)			<0.001
None	267 (16.3)	98 (25.7)	
Basic	896 (54.8)	213 (55.9)	
Secondary	299 (18.3)	51 (13.4)	
University	173 (10.6)	19 (5.0)	
Income level (%)			0.005
Till 1 salary	922 (56.4)	252 (66.1)	
>1 to 3 salaries	554 (33.9)	103 (27.0)	
>3 to 5 salaries	82 (5.0)	16 (4.2)	
>5 salaries	77 (4.7)	10 (2.6)	

a Among current smokers only. Results are expressed as number of participants (column percentage) for categorical variables and mean ± standard deviation for continuous variables. Between-group comparisons performed using chi-square or Fisher’s exact test (§) for categorical variables and by student’s *t*-test for continuous variables.

**Table 6:** Multivariable association between sociodemographic characteristics and recommendations provided reported by adult participants diagnosed with hypertension, brazilian national health survey 2019.

	Healthy eating	P-value	Healthy weight	P-value	Physical activity	P-value	Stop smoking^a^	P-value
Women	1.07 (0.98–1.18)	0.143	1.09 (1.00–1.19)	0.063	1.11 (1.02–1.21)	0.011	0.93 (0.73–1.17)	0.531
Age (per decade)	0.95 (0.92–0.99)	0.014	0.92 (0.89–0.96)	<0.001	0.82 (0.80–0.85)	<0.001	0.97 (0.88–1.08)	0.583
Ethnicity								
White	1 (ref)		1 (ref)		1 (ref)		1 (ref)	
Black	1.28 (1.10–1.48)	0.001	1.20 (1.04–1.37)	0.010	1.18 (1.04–1.34)	0.010	1.11 (0.77–1.61)	0.568
Asian	1.03 (0.62–1.73)	0.898	0.93 (0.58–1.47)	0.743	1.07 (0.68–1.68)	0.773	0.48 (0.17–1.30)	0.147
Mixed	1.08 (0.98–1.19)	0.139	1.03 (0.94–1.13)	0.514	1.05 (0.97–1.15)	0.237	0.90 (0.69–1.18)	0.446
Native	1.62 (0.89–2.95)	0.114	1.44 (0.85–2.45)	0.177	1.04 (0.67–1.61)	0.872	0.74 (0.20–2.71)	0.649
Marital status								
Married	1 (ref)		1 (ref)		1 (ref)		1 (ref)	
Divorced	0.91 (0.78–1.07)	0.259	0.83 (0.72–0.96)	0.012	0.88 (0.77–1.01)	0.063	1.20 (0.81–1.79)	0.369
Widowed	1.06 (0.93–1.21)	0.396	0.96 (0.85–1.09)	0.537	0.90 (0.80–1.00)	0.056	0.92 (0.64–1.32)	0.643
Single	0.84 (0.75–0.94)	0.002	0.81 (0.74–0.90)	<0.001	0.82 (0.75–0.91)	<0.001	1.01 (0.76–1.33)	0.956
Smoking status								
Never	1 (ref)		1 (ref)		1 (ref)		NC	
Former	0.80 (0.73–0.89)	<0.001	0.82 (0.75–0.90)	<0.001	0.95 (0.87–1.03)	0.195	NC	
Current	0.59 (0.52–0.68)	<0.001	0.59 (0.52–0.67)	<0.001	0.68 (0.60–0.77)	<0.001	NC	
Educational level								
None	0.71 (0.60–0.83)	<0.001	0.59 (0.51–0.69)	<0.001	0.47 (0.41–0.53)	<0.001	0.53 (0.35–0.82)	0.004
Basic	1 (ref)		1 (ref)		1 (ref)		1 (ref)	
Secondary	0.77 (0.68–0.88)	<0.001	0.74 (0.66–0.83)	<0.001	0.68 (0.60–0.76)	<0.001	0.76 (0.54–1.07)	0.120
University	1.06 (0.88–1.29)	0.521	1.11 (0.92–1.33)	0.267	1.33 (1.11–1.60)	0.002	1.57 (0.86–2.86)	0.144
P for trend	<0.001		<0.001		<0.001		<0.001	
Income level								
Till 1 salary	1 (ref)		1 (ref)		1 (ref)		1 (ref)	
>1 to 3 salaries	1.21 (1.10–1.34)	<0.001	1.20 (1.10–1.32)	<0.001	1.28 (1.18–1.39)	<0.001	1.29 (0.99–1.69)	0.059
>3 to 5 salaries	1.55 (1.23–1.95)	<0.001	1.71 (1.38–2.13)	<0.001	1.74 (1.42–2.13)	<0.001	0.92 (0.49–1.71)	0.789
>5 salaries	1.65 (1.27–2.16)	<0.001	1.78 (1.38–2.29)	<0.001	2.11 (1.64–2.71)	<0.001	1.22 (0.57–2.61)	0.613
P for trend	<0.001		<0.001		<0.001		0.842	

a Among current smokers only. NA, not available; NC, not considered. Results are expressed as odds ratio and (95% confidence interval) for a positive answer regarding the provision of the recommendation. Individual odds ratios are adjusted on all other variables of the table. Statistical analysis by logistic regression.

## 4 Discussion

Most Brazilians diagnosed with high cholesterol, diabetes or hypertension are given lifestyle recommendations, the most frequent being healthy eating. Still, and contrary to our initial hypothesis, the recommendations are unevenly distributed between educational and income categories, as better-educated, wealthier Brazilians reported receiving more lifestyle recommendations regarding high cholesterol and hypertension management than lower-educated or with low-income.

In this study, over four-fifths of participants reporting being diagnosed with high cholesterol, diabetes, or hypertension reported having received lifestyle recommendations, a value much higher than reported in European countries (Kotseva et al., 2021). This suggests that the Brazilian health system promotes non-medical management of cardiovascular risk factors prior to drug therapy, a strategy in agreement with international guidelines (Visseren et al., 2021).

### 4.1 Factors associated with provision of lifestyle recommendations for cardiovascular risk factors

Risk factors for CVD include modifiable metabolic and behavioural ones, which play a large role and include an unhealthy diet (e.g., lack of fruits and vegetables), physical inactivity, tobacco use, and alcohol consumption (Marques-Vidal, 2020, Visseren et al., 2021, Blumenthal et al., 2021).

Participants with higher socioeconomic and educational level had a higher likelihood of receiving lifestyle recommendations regarding healthy eating, healthy weight, and physical activities. Indeed, physical inactivity (Cruz et al., 2019, Da Silva et al., 2023), and unhealthy diet (Boing et al., 2019) were more common seen in low socioeconomic populations in Brazil according to the recent studies, while overweight and obesity were more common in high socioeconomic groups (Coimbra et al., 2021).

The reason for a lower provision of healthy eating and physical activity recommendations to participants of lower SES or lower income could be because people in those groups cannot afford to buy nutrient-rich foods, as these foods are more expensive than ultraprocessed, nutrient-poor foods (Mendoza-Velazquez et al., 2022). They could also be explained by the fact that most exercise is performed in paying facilities, due to the decline in availability of public spaces and awareness about public programs (Wendt et al., 2013). Overall, our results suggest that participants with low SES receive less recommendations regarding lifestyle prevention of cardiovascular risk factors. This lower provision could partly explain the higher level of high cholesterol levels (Malta et al., 2019) diabetes (Moradpour et al., 2022), diabetic complications (Tatulashvili et al., 2020) and hypertension (Dulgheroff et al., 2021) in this group. On the other hand, we cannot exclude the possibility that participants participants with a higher education may be more likely to report receiving recommendations, because they are more health-conscious and/or remember better the recommendations received than participants with low education (Hu et al., 2019). As participants with low education also have a lower income, their attention might be focused on other priorities than on the guidance provided by health professionals. Therefore, strategies and interventions for the disease prevention and control are highly recommended to prioritize this socio-economically disadvantaged group (Wang et al., 2022).

### 4.2 Strengths and limitations

This study has several strengths. First, it was performed on a national, representative sample. Second, the large sample size allowed to conduct detailed analyses of the individual effect of different socioeconomic markers on lifestyle recommendations regarding management of cardiovascular risk factors.

This study also has several limitations worth acknowledging. First, it was conducted in a South Latin American country, and its findings might not be generalizable to other settings. Still, similar findings on health inequalities regarding lifestyle have been reported in developed countries (Ding et al., 2015, Stait and Calnan, 2016). Second, the diagnosis of risk factors was self-reported and not based on objective measurements. Still, this is a common procedure among national health surveys, as drawing blood or measuring blood pressure would be logistically challenging in a country the size of Brazil and would likely reduce participation rates. Third, we don’t have information about who received the recommendations, only who reported receiving recommendations. Finally, a selection bias cannot be excluded, more health-conscious people being more likely to participate; this would overestimate the frequency of recommendations, but not the association between the participants’ characteristics and the provision of recommendations.

## 5 Conclusion

Better-educated, wealthier Brazilians reported receiving more lifestyle recommendations regarding high cholesterol and hypertension management than lower-educated or low-income population. Therefore, strategies and interventions for the disease prevention and control are highly recommended to prioritize those disadvantaged groups.

## Funding

This study was not funded.

## CRediT authorship contribution statement

**Pollyanna Patriota:** Conceptualization, Investigation, Writing – original draft, Visualization. **Ko Ko Maung:** Investigation, Writing – original draft. **Pedro Marques-Vidal:** Data curation, Formal analysis, Supervision, Writing – review & editing.

## Declaration of competing interest

The authors declare that they have no known competing financial interests or personal relationships that could have appeared to influence the work reported in this paper.

## Data Availability

The authors do not have permission to share data.
