# Supplementary material for: Reported recommendations to address cardiovascular risk factors differ by socio-economic status in Brazil. Results from the Brazilian National Health Survey 2019
Source: Prev Med Rep. 2023 Nov 25;36:102527. doi: 10.1016/j.pmedr.2023.102527 (PMC10728434; doi:10.1016/j.pmedr.2023.102527)
Supplement: Supplementary data 1 [file mmc1.docx]

**Supplementary information**

**Supplementary table 1:** original questions for diagnosis of cardiovascular risk factors, Brazilian National Health Survey 2019.

| **Original question (in Brazilian Portuguese)** | **Risk factor /**  **management** |
| --- | --- |
| **Diagnosis** |  |
| Algum médico já lhe deu o diagnóstico de colesterol alto? | Dyslipidemia |
| Algum médico já lhe deu o diagnóstico de diabetes? | Diabetes |
| Algum médico já lhe deu o diagnóstico de hipertensão arterial (pressão alta)? | Hypertension |
| **Management of high cholesterol** |  |
| Recomendação para manter uma alimentação saudável | Healthy eating |
| Recomendação para manter o peso adequado | Healthy weight |
| Recomendação para praticar atividade física regular | Physical activity |
| Recomendação para não fumar | Quitting smoking |
| **Management of diabetes** |  |
| Orientações para manter uma alimentação saudável | Healthy eating |
| Manter o peso adequado | Healthy weight |
| Praticar atividade física regular | Physical activity |
| Não fumar | Quitting smoking |
| **Management of hypertension** |  |
| Orientações para manter uma alimentação saudável | Healthy eating |
| Manter o peso adequado | Healthy weight |
| Praticar atividade física regular | Physical activity |
| Não fumar | Quitting smoking |

**Supplementary table 2**: characteristics of included and excluded participants, Brazilian National Health Survey 2019.

|  | **Included** | **Excluded** | **P-value** |
| --- | --- | --- | --- |
| N | 88,052 | 191,330 |  |
| Women (%) | 46,195 (52.5) | 98,745 (51.6) | <0.001 |
| Age (years) | 46.7 ± 17.6 | 30.0 ± 21.4 | <0.001 |
| Ethnicity |  |  | <0.001 |
| White | 32,308 (36.7) | 66,711 (34.9) |  |
| Black | 9,980 (11.3) | 18,324 (9.6) |  |
| Asian | 664 (0.8) | 1,034 (0.5) |  |
| Mixed | 44,448 (50.5) | 103,825 (54.3) |  |
| Native | 652 (0.7) | 1,412 (0.7) |  |
| Marital status |  |  | <0.001 |
| Single | 38,937 (44.2) | 94,814 (61.4) |  |
| Married | 34,314 (39) | 49,000 (31.7) |  |
| Divorced | 7,334 (8.3) | 5,486 (3.6) |  |
| Widowed | 7,467 (8.5) | 5,092 (3.3) |  |
| Educational level |  |  | <0.001 |
| None | 7,469 (8.5) | 17,780 (10.2) |  |
| Basic | 34,527 (39.2) | 80,777 (46.6) |  |
| Secondary | 28,825 (32.7) | 50,233 (29.0) |  |
| University | 17,231 (19.6) | 24,753 (14.2) |  |
| Smoking categories |  |  | <0.001 |
| Never | 53,509 (60.8) | 1,727 (61.8) |  |
| Former | 23,604 (26.8) | 620 (22.2) |  |
| Current | 10,939 (12.4) | 447 (16.0) |  |
| Income level (%) |  |  | <0.001 |
| Till 1 salary | 48,049 (54.6) | 125,679 (65.8) |  |
| >1 to 3 salaries | 29,483 (33.5) | 51,529 (27.0) |  |
| >3 to 5 salaries | 5,480 (6.2) | 7,900 (4.1) |  |
| >5 salaries | 5,040 (5.7) | 6,050 (3.2) |  |

Results are expressed as number of participants (column percentage) for categorical variables and mean ± standard deviation for continuous variables. Between-group comparisons performed using chi-square for categorical variables and by student’s t-test for continuous variables.

**Supplementary table 3**: association between socio-economic status and recommendations provided to participants diagnosed with high cholesterol, using inverse probability weighting to account for exclusions, Brazilian National Health Survey 2019.

|  | **Healthy eating** | **P-value** | **Healthy weight** | **P-value** | **Physical activity** | **P-value** | **Stop smoking †** | **P-value** |
| --- | --- | --- | --- | --- | --- | --- | --- | --- |
| Women | 0.95 (0.80 - 1.13) | 0.556 | 0.89 (0.78 - 1.01) | 0.065 | 1.05 (0.93 - 1.18) | 0.410 | 0.97 (0.74 - 1.28) | 0.838 |
| Age (per decade) | 0.90 (0.85 - 0.96) | 0.001 | 1.00 (0.95 - 1.05) | 0.946 | 0.83 (0.79 - 0.87) | <0.001 | 1.23 (1.09 - 1.39) | 0.001 |
| Ethnicity |  |  |  |  |  |  |  |  |
| White | 1 (ref) |  | 1 (ref) |  | 1 (ref) |  | 1 (ref) |  |
| Black | 1.38 (1.04 - 1.83) | 0.027 | 1.53 (1.24 - 1.89) | <0.001 | 1.19 (0.99 - 1.43) | 0.071 | 1.61 (1.04 - 2.49) | 0.032 |
| Asian | 0.56 (0.29 - 1.08) | 0.083 | 0.82 (0.46 - 1.46) | 0.507 | 1.34 (0.69 - 2.60) | 0.389 | 1.05 (0.27 - 4.11) | 0.945 |
| Mixed | 1.02 (0.86 - 1.21) | 0.821 | 1.12 (0.99 - 1.26) | 0.077 | 0.99 (0.88 - 1.11) | 0.879 | 1.04 (0.78 - 1.39) | 0.787 |
| Native | 1.40 (0.51 - 3.85) | 0.519 | 1.70 (0.77 - 3.75) | 0.185 | 0.90 (0.49 - 1.65) | 0.734 | 0.66 (0.20 - 2.18) | 0.499 |
| Marital status |  |  |  |  |  |  |  |  |
| Married | 1 (ref) |  | 1 (ref) |  | 1 (ref) |  | 1 (ref) |  |
| Divorced | 0.88 (0.68 - 1.13) | 0.309 | 0.90 (0.74 - 1.09) | 0.281 | 0.86 (0.71 - 1.03) | 0.109 | 1.00 (0.66 - 1.53) | 0.992 |
| Widowed | 1.14 (0.89 - 1.45) | 0.295 | 0.99 (0.82 - 1.19) | 0.928 | 0.84 (0.72 - 0.99) | 0.037 | 0.89 (0.57 - 1.40) | 0.624 |
| Single | 0.84 (0.69 - 1.01) | 0.067 | 0.72 (0.63 - 0.83) | <0.001 | 0.73 (0.64 - 0.83) | <0.001 | 0.85 (0.63 - 1.16) | 0.317 |
| Smoking status |  |  |  |  |  |  |  |  |
| Never | 1 (ref) |  | 1 (ref) |  | 1 (ref) |  | NC |  |
| Former | 0.91 (0.77 - 1.06) | 0.228 | 0.93 (0.82 - 1.05) | 0.254 | 1.00 (0.90 - 1.13) | 0.935 | NC |  |
| Current | 0.71 (0.56 - 0.90) | 0.005 | 0.72 (0.61 - 0.87) | <0.001 | 0.76 (0.64 - 0.90) | 0.002 | NC |  |
| Educational level |  |  |  |  |  |  |  |  |
| None | 0.68 (0.51 - 0.90) | 0.008 | 0.63 (0.51 - 0.77) | <0.001 | 0.41 (0.33 - 0.50) | <0.001 | 0.42 (0.25 - 0.68) | <0.001 |
| Basic | 1 (ref) |  | 1 (ref) |  | 1 (ref) |  | 1 (ref) |  |
| Secondary | 0.67 (0.55 - 0.83) | <0.001 | 0.71 (0.61 - 0.83) | <0.001 | 0.54 (0.46 - 0.63) | <0.001 | 0.70 (0.49 - 0.99) | 0.050 |
| University | 0.89 (0.67 - 1.19) | 0.433 | 0.95 (0.78 - 1.17) | 0.645 | 1.06 (0.85 - 1.33) | 0.586 | 0.78 (0.48 - 1.27) | 0.317 |
| P for trend | 0.027 |  | <0.001 |  | <0.001 |  | 0.019 |  |
| Income level |  |  |  |  |  |  |  |  |
| Till 1 salary | 1 (ref) |  | 1 (ref) |  | 1 (ref) |  | 1 (ref) |  |
| >1 to 3 salaries | 1.35 (1.14 - 1.62) | 0.001 | 1.20 (1.06 - 1.37) | 0.006 | 1.26 (1.12 - 1.43) | <0.001 | 1.06 (0.79 - 1.43) | 0.698 |
| >3 to 5 salaries | 1.74 (1.20 - 2.52) | 0.004 | 1.59 (1.21 - 2.08) | 0.001 | 1.60 (1.22 - 2.08) | 0.001 | 1.28 (0.67 - 2.44) | 0.449 |
| >5 salaries | 1.55 (1.05 - 2.29) | 0.028 | 1.84 (1.36 - 2.50) | <0.001 | 1.67 (1.24 - 2.24) | 0.001 | 1.83 (0.90 - 3.73) | 0.095 |
| P for trend | 0.016 |  | <0.001 |  | <0.001 |  | 0.093 |  |

**†** among current smokers only. NC, not considered. Results are expressed as odds ratio and (95% confidence interval). Statistical analysis by logistic regression.

**Supplementary table 4**: association between socio-economic status and recommendations provided to participants diagnosed with diabetes, using inverse probability weighting to account for exclusions, Brazilian National Health Survey 2019.

|  | **Healthy eating** | **P-value** | **Healthy weight** | **P-value** | **Physical activity** | **P-value** | **Stop smoking †** | **P-value** |
| --- | --- | --- | --- | --- | --- | --- | --- | --- |
| Women | 0.88 (0.69 - 1.13) | 0.323 | 0.97 (0.79 - 1.18) | 0.728 | 0.95 (0.81 - 1.12) | 0.561 | 0.54 (0.33 - 0.90) | 0.018 |
| Age (per decade) | 0.86 (0.77 - 0.96) | 0.007 | 0.84 (0.76 - 0.92) | <0.001 | 0.70 (0.65 - 0.76) | <0.001 | 1.06 (0.84 - 1.33) | 0.635 |
| Ethnicity |  |  |  |  |  |  |  |  |
| White | 1 (ref) |  | 1 (ref) |  | 1 (ref) |  | 1 (ref) |  |
| Black | 1.15 (0.79 - 1.68) | 0.468 | 1.14 (0.83 - 1.57) | 0.409 | 1.34 (1.05 - 1.72) | 0.020 | 2.73 (1.03 - 7.27) | 0.044 |
| Asian | 1.52 (0.37 - 6.32) | 0.565 | 0.86 (0.33 - 2.24) | 0.754 | 2.96 (0.88 - 9.93) | 0.079 | NC |  |
| Mixed | 0.97 (0.75 - 1.24) | 0.788 | 0.97 (0.79 - 1.20) | 0.807 | 1.16 (0.98 - 1.36) | 0.077 | 1.05 (0.62 - 1.77) | 0.856 |
| Native | 1.50 (0.36 - 6.27) | 0.575 | 4.64 (0.63 - 34.4) | 0.133 | 1.77 (0.67 - 4.67) | 0.246 | 0.43 (0.09 - 2.08) | 0.291 |
| Marital status |  |  |  |  |  |  |  |  |
| Married | 1 (ref) |  | 1 (ref) |  | 1 (ref) |  | 1 (ref) |  |
| Divorced | 0.75 (0.52 - 1.08) | 0.119 | 0.89 (0.65 - 1.23) | 0.481 | 0.98 (0.76 - 1.28) | 0.903 | 1.5 (0.69 - 3.25) | 0.301 |
| Widowed | 0.94 (0.68 - 1.30) | 0.709 | 1.05 (0.81 - 1.37) | 0.714 | 0.95 (0.78 - 1.17) | 0.646 | 1.86 (0.86 - 4.00) | 0.114 |
| Single | 0.77 (0.58 - 1.04) | 0.090 | 0.79 (0.62 - 1.01) | 0.058 | 0.82 (0.67 - 0.99) | 0.044 | 1.35 (0.75 - 2.41) | 0.317 |
| Smoking status |  |  |  |  |  |  |  |  |
| Never | 1 (ref) |  | 1 (ref) |  | 1 (ref) |  | NC |  |
| Former | 0.86 (0.68 - 1.10) | 0.230 | 0.94 (0.77 - 1.14) | 0.523 | 0.86 (0.74 - 1.00) | 0.056 | NC |  |
| Current | 0.77 (0.53 - 1.13) | 0.183 | 0.69 (0.51 - 0.94) | 0.019 | 0.72 (0.56 - 0.93) | 0.013 | NC |  |
| Educational level |  |  |  |  |  |  |  |  |
| None | 0.7 (0.47 - 1.06) | 0.089 | 0.55 (0.4 - 0.77) | <0.001 | 0.47 (0.36 - 0.62) | <0.001 | 0.89 (0.40 - 1.96) | 0.772 |
| Basic | 1 (ref) |  | 1 (ref) |  | 1 (ref) |  | 1 (ref) |  |
| Secondary | 0.76 (0.55 - 1.06) | 0.108 | 0.77 (0.58 - 1.01) | 0.063 | 0.66 (0.53 - 0.83) | <0.001 | 1.21 (0.65 - 2.26) | 0.548 |
| University | 0.82 (0.49 - 1.35) | 0.431 | 0.83 (0.54 - 1.28) | 0.393 | 0.94 (0.65 - 1.35) | 0.723 | 1.04 (0.39 - 2.79) | 0.939 |
| P for trend | 0.399 |  | 0.037 |  | <0.001 |  | 0.877 |  |
| Income level |  |  |  |  |  |  |  |  |
| Till 1 salary | 1 (ref) |  | 1 (ref) |  | 1 (ref) |  | 1 (ref) |  |
| >1 to 3 salaries | 1.48 (1.15 - 1.91) | 0.002 | 1.36 (1.10 - 1.67) | 0.004 | 1.29 (1.10 - 1.52) | 0.002 | 1.08 (0.62 - 1.87) | 0.789 |
| >3 to 5 salaries | 1.65 (0.96 - 2.84) | 0.070 | 1.70 (1.08 - 2.68) | 0.023 | 1.96 (1.37 - 2.82) | <0.001 | 1.47 (0.36 - 6.06) | 0.595 |
| >5 salaries | 1.53 (0.83 - 2.84) | 0.175 | 1.63 (0.95 - 2.80) | 0.078 | 2.22 (1.40 - 3.51) | 0.001 | 1.66 (0.41 - 6.60) | 0.475 |
| P for trend | 0.175 |  | 0.059 |  | <0.001 |  | 0.426 |  |

**†** among current smokers only. NA, not available; NC, not considered. Results are expressed as odds ratio and (95% confidence interval). Statistical analysis by logistic regression.

**Supplementary table 5**: association between socio-economic status and recommendations provided to participants diagnosed with high blood pressure, using inverse probability weighting to account for exclusions, Brazilian National Health Survey 2019.

|  | **Healthy eating** | **P-value** | **Healthy weight** | **P-value** | **Physical activity** | **P-value** | **Stop smoking †** | **P-value** |
| --- | --- | --- | --- | --- | --- | --- | --- | --- |
| Women | 1.07 (0.98 - 1.18) | 0.138 | 1.09 (1.00 - 1.19) | 0.060 | 1.11 (1.02 - 1.21) | 0.011 | 0.93 (0.73 - 1.17) | 0.526 |
| Age (per decade) | 0.96 (0.92 - 0.99) | 0.023 | 0.92 (0.89 - 0.96) | <0.001 | 0.83 (0.80 - 0.85) | <0.001 | 0.97 (0.88 - 1.08) | 0.601 |
| Ethnicity |  |  |  |  |  |  |  |  |
| White | 1 (ref) |  | 1 (ref) |  | 1 (ref) |  | 1 (ref) |  |
| Black | 1.28 (1.10 - 1.48) | 0.001 | 1.20 (1.04 - 1.37) | 0.010 | 1.18 (1.04 - 1.34) | 0.010 | 1.11 (0.77 - 1.60) | 0.577 |
| Asian | 1.04 (0.62 - 1.74) | 0.886 | 0.93 (0.58 - 1.49) | 0.765 | 1.07 (0.68 - 1.68) | 0.766 | 0.48 (0.19 - 1.19) | 0.113 |
| Mixed | 1.08 (0.98 - 1.19) | 0.132 | 1.03 (0.94 - 1.13) | 0.495 | 1.05 (0.97 - 1.15) | 0.234 | 0.90 (0.68 - 1.18) | 0.445 |
| Native | 1.62 (0.89 - 2.95) | 0.117 | 1.44 (0.85 - 2.45) | 0.180 | 1.04 (0.67 - 1.60) | 0.864 | 0.75 (0.21 - 2.71) | 0.659 |
| Marital status |  |  |  |  |  |  |  |  |
| Married | 1 (ref) |  | 1 (ref) |  | 1 (ref) |  | 1 (ref) |  |
| Divorced | 0.91 (0.78 - 1.07) | 0.256 | 0.83 (0.72 - 0.96) | 0.012 | 0.88 (0.77 - 1.01) | 0.064 | 1.20 (0.81 - 1.78) | 0.365 |
| Widowed | 1.06 (0.92 - 1.21) | 0.417 | 0.96 (0.85 - 1.09) | 0.524 | 0.90 (0.80 - 1.00) | 0.055 | 0.92 (0.64 - 1.32) | 0.636 |
| Single | 0.84 (0.75 - 0.94) | 0.002 | 0.81 (0.74 - 0.90) | <0.001 | 0.82 (0.75 - 0.91) | <0.001 | 1.01 (0.76 - 1.33) | 0.964 |
| Smoking status |  |  |  |  |  |  |  |  |
| Never | 1 (ref) |  | 1 (ref) |  | 1 (ref) |  | NC |  |
| Former | 0.80 (0.73 - 0.89) | <0.001 | 0.82 (0.75 - 0.90) | <0.001 | 0.95 (0.87 - 1.03) | 0.199 | NC |  |
| Current | 0.59 (0.52 - 0.68) | <0.001 | 0.59 (0.52 - 0.67) | <0.001 | 0.68 (0.60 - 0.77) | <0.001 | NC |  |
| Educational level |  |  |  |  |  |  |  |  |
| None | 0.71 (0.60 - 0.83) | <0.001 | 0.59 (0.51 - 0.69) | <0.001 | 0.47 (0.41 - 0.54) | <0.001 | 0.53 (0.35 - 0.82) | 0.004 |
| Basic | 1 (ref) |  | 1 (ref) |  | 1 (ref) |  | 1 (ref) |  |
| Secondary | 0.77 (0.68 - 0.88) | <0.001 | 0.74 (0.66 - 0.83) | <0.001 | 0.68 (0.60 - 0.76) | <0.001 | 0.76 (0.54 - 1.08) | 0.126 |
| University | 1.07 (0.88 - 1.29) | 0.509 | 1.11 (0.92 - 1.34) | 0.270 | 1.34 (1.11 - 1.61) | 0.002 | 1.56 (0.84 - 2.91) | 0.157 |
| P for trend | <0.001 |  | <0.001 |  | <0.001 |  | <0.001 |  |
| Income level |  |  |  |  |  |  |  |  |
| Till 1 salary | 1 (ref) |  | 1 (ref) |  | 1 (ref) |  | 1 (ref) |  |
| >1 to 3 salaries | 1.21 (1.10 - 1.34) | <0.001 | 1.20 (1.09 - 1.32) | <0.001 | 1.28 (1.17 - 1.39) | <0.001 | 1.29 (0.99 - 1.69) | 0.064 |
| >3 to 5 salaries | 1.55 (1.23 - 1.95) | <0.001 | 1.71 (1.37 - 2.13) | <0.001 | 1.74 (1.42 - 2.13) | <0.001 | 0.92 (0.50 - 1.68) | 0.778 |
| >5 salaries | 1.65 (1.26 - 2.16) | <0.001 | 1.78 (1.37 - 2.31) | <0.001 | 2.1 (1.64 - 2.7) | <0.001 | 1.21 (0.53 - 2.76) | 0.648 |
| P for trend | <0.001 |  | <0.001 |  | <0.001 |  | 0.864 |  |

**†** among current smokers only. NA, not available; NC, not considered. Results are expressed as odds ratio and (95% confidence interval). Statistical analysis by logistic regression.
